# Resistance to Antiandrogens in Prostate Cancer: Is It Inevitable, Intrinsic or Induced?

**DOI:** 10.3390/cancers13020327

**Published:** 2021-01-17

**Authors:** Norman J. Maitland

**Affiliations:** Department of Biology, University of York, Heslington, York YO10 5DD, UK; n.j.maitland@york.ac.uk

**Keywords:** prostate cancer, androgens, androgen deprivation therapy: tumor resistance, model systems

## Abstract

**Simple Summary:**

Biochemical inhibition of male sex hormone function (androgen signaling), also known as androgen deprivation therapy (ADT), for human prostate cancer remains a major treatment strategy almost 80 years after the discovery of androgens as a major factor in the disease. Drug development has resulted in an increasing potency, whereas the understanding of the consequences of these new-generation inhibitors in cancer survivors for increased periods of time, and indeed for their individual cancer cells, has lagged behind. Drugs are still tested in laboratory cell systems developed 40 years ago, which indicate a toxic effect of the antiandrogens on the tumor cells, not matched by direct studies of human tissues. In this review, I discuss the limits of our understanding of both how these drugs work and potential side effects, which are often overlooked in the face of a perceived urgency to get better inhibitors in to the clinic.

**Abstract:**

Increasingly sophisticated therapies for chemical castration dominate first-line treatments for locally advanced prostate cancer. However, androgen deprivation therapy (ADT) offers little prospect of a cure, as resistant tumors emerge rather rapidly, normally within 30 months. Cells have multiple mechanisms of resistance to even the most sophisticated drug regimes, and both tumor cell heterogeneity in prostate cancer and the multiple salvage pathways result in castration-resistant disease related genetically to the original hormone-naive cancer. The timing and mechanisms of cell death after ADT for prostate cancer are not well understood, and off-target effects after long-term ADT due to functional extra-prostatic expression of the androgen receptor protein are now increasingly being recorded. Our knowledge of how these widely used treatments fail at a biological level in patients is deficient. In this review, I will discuss whether there are pre-existing drug-resistant cells in a tumor mass, or whether resistance is induced/selected by the ADT. Equally, what is the cell of origin of this resistance, and does it differ from the treatment-naïve tumor cells by differentiation or dedifferentiation? Conflicting evidence also emerges from studies in the range of biological systems and species employed to answer this key question. It is only by improving our understanding of this aspect of treatment and not simply devising another new means of androgen inhibition that we can improve patient outcomes.

## 1. Introduction

**Figure 1:**
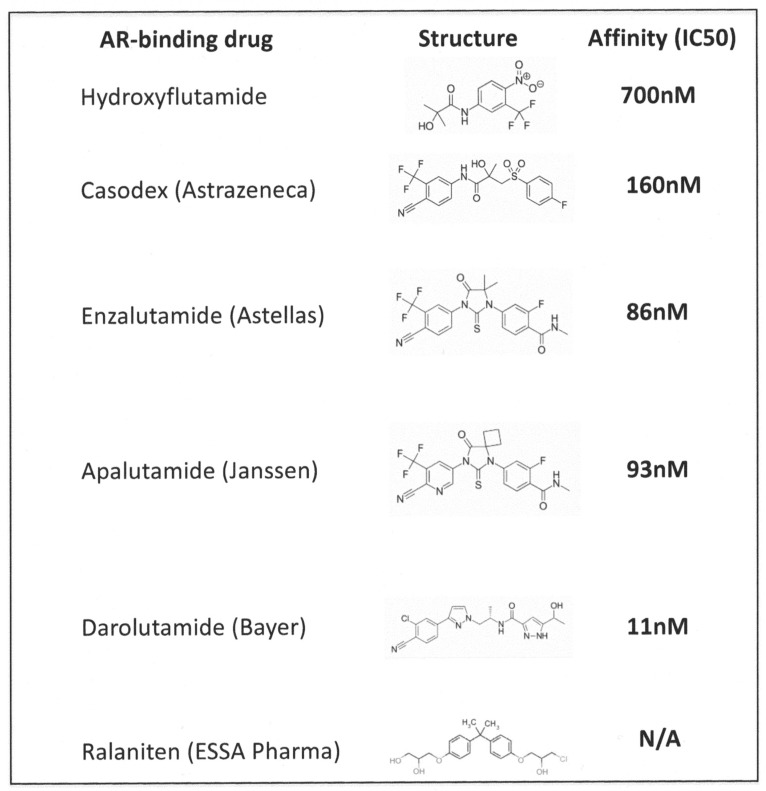
The evolution of direct (nonsteroidal) inhibitors of the androgen receptor. The increase in affinity of the drugs for the receptor with structural engineering is striking [4]. Note also that darolutamide is stated not to cross the blood–brain barrier, unlike the other molecules, and ralaniten binds to a different part (the flexible portion of the Androgen Receptor (AR) molecule, so a comparable IC50 is not appropriate. Ralaniten has recently been withdrawn due to low clinical efficacy, despite promising preclinical data).

Treatment protocols for prostate cancers have developed in the years since the Nobel prize was awarded to Charles Huggins in 1966 for the demonstration that hormone manipulation via orchidectomy results in the remission of hormone-sensitive prostate cancer [1]. The current use of chemical castration agents is more acceptable than surgery to most patients, and the pharmaceutical industry has invested considerably in the development of various generations of these drugs, such as enzalutamide and darolutamide (Figure 1). These drugs are powerful inhibitors which bind with differing affinities to the androgen receptor protein and their clinical application is constantly being reviewed. The structure of androgen response inhibitors originally mimicked testosterone (steroidal antiandrogens), but current nonsteroidal antiandrogens are molecularly optimized for maximal inhibitory effects.

Improvements in the drugs’ affinities for the Androgen Receptor (AR) target (Figure 1) and the structural chemistry/molecular fitting approaches used for their development have resulted in improved biochemical properties in cell culture testing. However, the mean time from Androgen Deprivation Therapy (ADT) treatment start to relapse (assessed using increase in serum Prostate-Specific Antigen (PSA) levels) remains around 30 months for patients without metastases and half that for patients with metastases [2]. There is also some evidence that very high initial Gleason scores (9/10) of cancer progress more rapidly to castration-resistant disease after ADT than on placebo [3].

### 1.1. Androgen-Based Therapies Retain Activity after Failure of First-Line AR-Based Treatments

After the failure of first-line androgen deprivation therapy (ADT), such as luteinizing hormone-releasing hormone (LHRH) antagonists and bicalutamide, next-generation hormone-based therapies such as abiraterone can alter the intratumoral androgen production [5] that is observed in castration-resistant prostate cancer (CRPC) or enzalutamide [6] and apalutamide [7] can suppress residual androgen responses in CRPC [6]. After the (almost inevitable) failure of even the latter androgen-based treatments [8], less specific, replication-based, more toxic chemotherapies such taxane treatments are applied for castration-resistant prostate cancers. Some hope of further specific chemotherapies, such as olaparib, which target the proportion of prostate cancers with DNA damage repair defects recently showed promise in clinical trials [9]. At this point, the oncologist can do little beyond palliation to mitigate the development of the most fatal form of disease, with a poorly differentiated histology, typical of the higher Gleason grades. The advanced CRPC lesions display a basaloid or neuroendocrine phenotype (neuroendocrine prostate cancer—NEPC) and ultimately have a poor prognosis [10].

### 1.2. Combination Therapies Which Include Androgen Blockades Can Extend Patient Survival

Combination therapies, featuring both taxanes and androgen signaling inhibition, are improving survival in newly diagnosed patients with high-grade metastatic disease [11]. However, other novel immunotherapies have, to date, failed to show the same promise in prostate cancer treatment, with efficacy only in a minor population of patients [12], unlike the large-scale remissions seen in melanoma, small cell lung cancer, and some leukemias [13]. 

### 1.3. Androgen Blockade: A Time-Limited Treatment?

Nevertheless, ADT remains the mainstay of primary drug treatment for PCa. A patient selected for hormone therapy will normally derive benefits for 1–4 years, although in a small minority of patients, the remission can last for up to 10 years. The future of ADT seems to lie with the generation of new and improved androgen signaling inhibitors (Figure 1) [4], despite the expense incurred by both the pharmaceutical industry and the inevitable extra costs to healthcare systems. Combination treatments, and various total androgen blockades—both constant and intermittent [14]—have all been tested in clinical trials. However, the cancer returns despite apparent increased survival in intermittently treated populations [15], a therapeutic strategy that is probably underused. If the tumor cells are indeed dependent on androgens, even in CRPC [16], then why a cocktail of inhibitors of androgen signaling does not inevitably result in increased remission or even a cure is unclear. 

The use of new androgen receptor inhibitor strategies is probably also fueled by the size of the worldwide markets, which was USD 7 billion in 2019 (according to alliedmarketresearch.com). This market is set to increase by a rate of >10% every year, driven by a combination of increasing patient numbers in the Western world and improved healthcare in countries where prostate cancer in elderly males was once a minor disease, due to historically shorter life spans as a result of mainly infectious diseases. 

In this review, I consider the biochemical and biological mechanisms of resistance, both obvious and more esoteric, in order to open a debate about how to optimize the application of the current best, but nevertheless time-limited, therapeutic strategy. I will examine the limits of our knowledge, focusing on the biological and molecular responses to prostate cancer treatments, which primarily inhibit the response to androgen.

## 2. Targeting the Androgen Signaling Pathway

**Figure 2:**
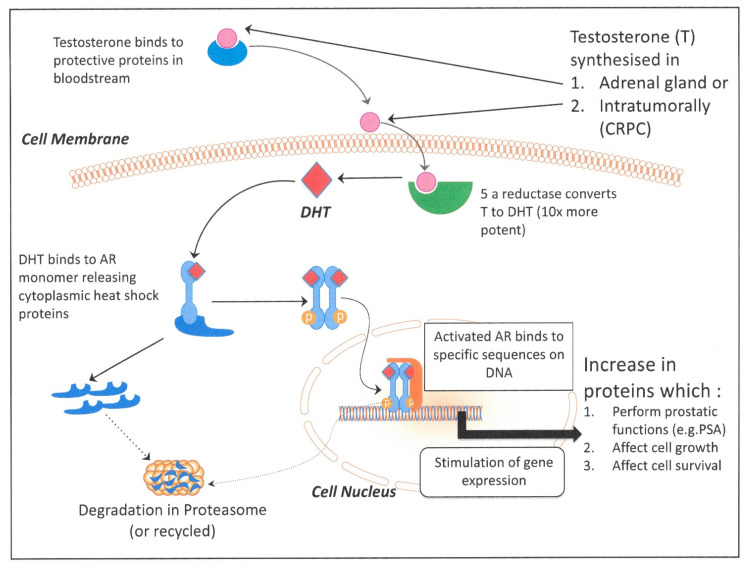
The androgen signaling cascade in prostate epithelial cells.

The inherent complexity and multistep nature of the androgen response pathway, and the tissue-specific molecules involved (Figure 2), show that this signaling pathway is an ideal therapeutic target, but increasing identification of functional androgen receptor (AR) expression in non-prostate tissues (see below, 10.2) could mitigate the utility of targeting this pathway. The cellular processes involved in the androgen response cascade that are targetable can be broken down into discrete therapeutic intervention points:

- Extracellular provision of testosterone;
- Activation of testosterone by 5α reductase to dihydrotestosterone (DHT);
- Androgen metabolism and receptor engagement in the cell cytoplasm;
- Turnover and metabolism of the AR and co-activator proteins;
- Transcription complex formation and activation of gene expression in the cell nucleus;
- Blockade of AR-stimulated genes and cytokines—second messengers

**Figure 3:**
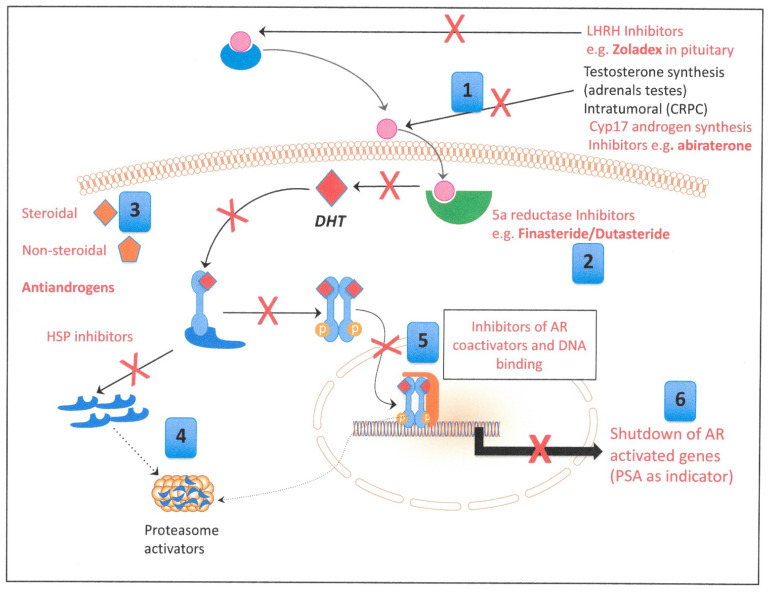
Known therapeutic interventions to block androgen signaling. Specific inhibitors shown in red. Blue boxes correspond to headline strategies in Table 1. HSP: Heat Shock Proteins, LHRH: Luteinizing hormone-releasing hormone, Cyp17: Cytochrome P450 17α−hydroxy/17,20-lyase, AR: Androgen Receptor, PSA: Prostate Specific Antigen, DHT: Dihydrotestosterone, CRPC: Castration Resistant Prostate Cancer.

A schematic view of the various therapeutic interventions that are currently employed is shown below (Figure 3). The molecular strategies can be divided into three main categories:

- Direct binding inhibitors of the AR;
- Testosterone activating 5α- reductase inhibitors and
- Intratumoral and extratumoral testosterone inhibitors.

Chemical inhibition has been achieved with a high degree of specificity and the biochemical consequences of these interventions have been described in detail elsewhere [17,18] and the referencing in Table 1.

### 2.1. Resistance to Androgen Blockade

The multiple alternative signaling mechanisms present in the AR-expressing cells is increasingly apparent. These mechanisms enable escape from ADT and maintenance of AR signaling in both normal and malignant cells (Table 1). For example, inhibition of androgen synthesis by gosrelin or abiraterone can be circumvented by intratumoral androgen synthesis and AR expression amplification or via the glucocorticoid receptor and glucocorticoids, respectively [19,20,21,22,23,24]. In evolutionary terms, the escape, salvage and/or backup signaling pathways are a useful resource for an affected cell, enabling the persistence of survival and proliferative signals in the absence of the principal ligand response. For a mammalian population, loss of androgen signaling would have extreme effects on fertility and reproduction, providing a strong evolutionary driving force for the presence of alternative salvage pathways.

### 2.2. Targeting Alternative Pathways 

**Figure 4:**
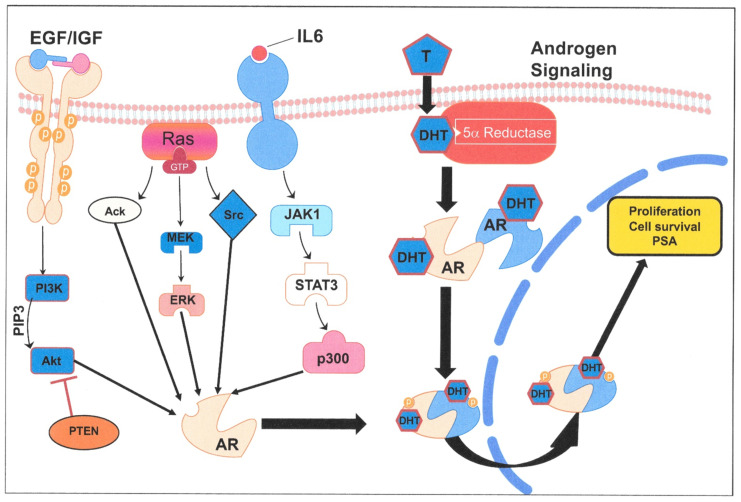
Alternative growth factor driven signaling pathways after androgen blockade. Canonical androgen response is shown on the right of the figure (as in Figure 3), whereas under conditions of limiting androgens or ADT, at least three alternative pathways can be activated, all resulting in steroid-independent activation of AR signaling: (i) Epidermal Growth Factor and Insulin-Like Growth Factor (EGF/IGF) stimulated signalling via Phosphatidylinositol 3-kinase (PI3K), Protein kinase B ( Akt/PKB) and mediated by phosphatidylinositol 3,4,5-triphosphate (PIP3) and Phosphatase and tensin homolog (PTEN) levels in cells. (ii) Signalling with the ras proto-oncogene (ras signalling) via Activated Cdc42-associated kinase (Ack), The Ras/Raf/Mitogen-activated protein kinase/ERK kinase (MEK) pathway and the Proto-oncogene tyrosine-protein kinase Src (Src), and (iii) Interleukin 6 (IL6) cytokine signalling which activartes AR via janus kinase-signal transducer and activator of transcription (JAK1), signal transducer and activator of transcription 3 (STAT3) and histone acetyltransferase p300 (p300) intermediates as shown.

In prostate cancer, a known number of alternative AR activation pathways exist, driven by cytokines and growth factors (Table 1 and Figure 4). In particular, the IL6 pathway has been a target for inhibitor trials, which were originally developed to target inflammatory responses. However, trials in prostate cancer did not result in considerable survival advantages, despite promising results in the initial phases [52,53]. Signaling via epidermal growth factor and insulin-like growth factor has also been shown to activate AR signaling [54], but the ubiquity of these pathways in normal tissue function probably means that the risks of adverse (and long-term) effects through targeting them are high. As the alternative pathways do not exploit particular gene mutations, they should pre-exist in androgen receptor inhibitor treatments and are almost certainly active in a number of other cell types that either lack exposure to circulating androgens or some of the other intermediate signaling molecules such as 5α reductase (Figure 3). Many of the growth-factor-stimulated pathways are constitutively activated by paracrine or autocrine secretion of and response to growth factors (such as TGFβ) either in tumor cells or in the contributing cells in the tumor microenvironment (for example, stromal and hematopoietic cells) [55,56]. TGFβ can have acutely toxic short-term effects on BPH1 cells [55], and persistent exposure to TGFβ has been shown to promote and increase tumorigenic properties, including epithelial to mesenchymal transition (EMT), in breast progenitor cells [57]. Most prostate tumor models used for therapeutic development both in vitro and in vivo (which were initially selected purely for their abilities to grow quickly) do not share the activation of these intercellular signaling pathways with human tumors *in vivo* and are therefore incomplete models.

The list of potential resistance mechanisms to ADT is long (Table 1) and ubiquitous for all proposed therapeutic strategies. Although some of these are druggable, there is a fundamental gap in our knowledge of when and how to anticipate resistance mechanisms. Again, the existence of a mechanism in vitro does not necessarily mean that it is functional in vivo. For example, a tumor consisting of several million cells could contain rare pre-existing cells that have activated drug resistance towards the development of CRPC (intrinsic resistance). Presumably, the larger the tumor size, or perhaps the existence of defects in DNA repair mechanisms, would increase the presence of such pre-existing resistant tumor clones. Does such increased tumor cell heterogeneity provide an explanation for the recently described differences in the efficacy of ADT in higher Gleason grade cancers [3]? Furthermore, tumor cells could undergo trans-differentiation or mutation in response to the treatment (induced resistance). This will be discussed in more detail below. Clearly, a successful treatment strategy should block the resistance mechanisms, but the method employed depends critically on which mechanism the tumor cell uses to escape ADT.

Novel resistance mechanisms are being uncovered with increased frequency as next-generation antiandrogen treatment fails [58,59]. In addition to the established ADT resistance mechanisms, such as AR gene amplification and splice variants, amplification of an AR transcriptional enhancer has been discovered which boosts the activation of AR-regulated genes even in the presence of ADT [31].

Metabolic changes in cells, such as increased and altered lipid usage, may also play a role in CRPC development [60], and redifferentiation or trans-differentiation of tumour cells to different cell types, such as cells with a gastrointestinal phenotype with a primitive stem-like transcriptome [61,62], has been observed. Increased expression of stem and embryonic master regulators [63] such as NOTCH [64] and WNT [65] has been reported in CRPC tissues after enzalutamide treatment, but this effect could be a post-treatment regenerative response rather than a true adaptation.

With so many known alternative pathways available to a prostate cancer cell seeking to bypass drug treatments, the time-limited responses to ADT based on AR signaling inhibitors is perhaps not surprising. Multiple redundancies of these salvage or alternative pathways also compounds the difficulty of achieving truly global inhibition of AR function by combinatorial therapy. In almost all situations, a resistant clone will emerge from a treated tumor mass if the adapted cells have a growth or survival advantage. The ideal life-extending combination treatment should therefore anticipate resistance, rather than be administered when the resistant cell type has emerged and could further adapt. Only a few early biomarkers of the emergence of resistance have been discovered (see Section 4) to guide such an anticipatory blockade and there is no certainty that several escape mechanisms are simultaneously employed in the treated tumor mass, with one likely to predominate, given the heterogeneity seen in patient studies.

## 3. Mechanisms of Cell Death after Application of Androgen Signaling Blockade

As the aim of ADT is to eliminate prostate cancers in man, it is important to know the precise mechanism of cell death induced by the drugs, if we wish to overcome resistance. Even after the length of time since the therapy was first applied clinically, the death mechanism remains unclear and is defined by the experimental model used for study. For example, does castration by surgical or chemical means actually induce cell death by apoptosis, or is the effect more passive: either senescence or atrophy?

### 3.1. The LNCaP Cell Line: Industry Standard Sndrogen-Responsive PCa Cell Model

Until fairly recently, the industry standard cell for ADT drug development was almost exclusively the LNCaP fast-growing clone (LNCaP-FGC), originally reported over 30 years ago [66]. The patient origin of the LNCaP cell lines is often misunderstood to be a well-differentiated cancer model for ADT. However, according to Horoszewicz et al., [66], “The patient was a 50-year old male with stage D1 prostate cancer. He was originally treated with oral estrogens, but 6 months later, bony metastases were detected. The patient was randomized and treated with methyl CCNU (a chemical carcinogen) and Estracyt (a dual estrogen and nitrogen mustard chemotherapy). One month later a supraclavicular lymph node was palpated and a needle aspiration confirmed the diagnosis of metastatic carcinoma. It was from this biopsy that the LNCaP culture was initiated”. Subsequently, a mutation (Codon 877 Thr to Ala) in the AR gene [67], which perhaps unsurprisingly, given the treatment regime, renders the LNCaP cells susceptible to growth stimulation by estrogens, was found [68]. The karyotype of the LNCaP cells is highly aneuploid, more reminiscent of CRPC than that of a primary cancer still treatable with androgen receptor inhibitors (2790 exome mutations in LNCaP vs. 16–33 in primary cancer hormone-naïve biopsy exomes) [69]. Intriguingly, LNCaP contained multiple X chromosomes (the gene locus for AR) in the original cell line [70], but a recent re-quantification of the AR copy number in LNCaP by my own laboratory has confirmed the single, mutated copy in the current ATCC reference culture. The CRPC characteristics of LNCaP were also reflected in the undifferentiated non-glandular tumor histology, both in man but also in the nude mouse xenograft tumors which appeared 16 weeks after inoculation of 10^7^ original LNCaP cells and were serially transplantable. Despite the origin in a metastatic lesion, LNCaP are only modestly invasive into local muscle from a subcutaneous graft [66]. Most studies confirm that LNCaP growth responds positively, if modestly, compared to its responsiveness to epidermal growth factor, to medium supplementation by dihydrotestosterone or estrogen [71], and its growth is inhibited by AR inhibitors such as bicalutamide [72]. The cells do express prodigious amounts of prostatic acid phosphatase and PSA, however, as evidence of positive AR signaling.

### 3.2. Cell Death in Cell Line Models of PCa after Androgen Signaling Blockade

Published evidence for increased cell death due to apoptosis after ADT by bicalutamide treatment is more modest. In one study, there was no increase in cleaved PARP, Caspase 3/7 or 8 after monotherapy of both LNCaP and 22RV1 cells with 10 micromolar bicalutamide [73]. In contrast, extensive treatment with 100 micromolar bicalutamide [74] did provide evidence of apoptotic death after 24–48 h treatment. However, the latter result, with the requirement for such a high concentration of bicalutamide, may be of limited relevance to a clinical treatment. It seems more likely that androgens protect LNCaP cells against their naturally high levels of apoptotic cell death (around 15% in any population —Dr M Sharrard, personal communication) and that inhibition of signaling merely restores this. One study of natural, rather than chemical, AR inhibition by culturing LNCaP cells under androgen-free conditions in vitro, and in both patient material and LNCaP xenografts in vivo, strongly suggested irreversible senescence rather than apoptotic cell death [73] as the mechanism for cell losses after castration. LNCaP cell cycle arrest, senescence and atrophy in response to bicalutamide, but not to the earlier steroidal anti-androgen flutamide, is more commonly reported [75]. In a further in vitro/in vivo study with the LNCaP model, treatment with Casodex or enzalutamide seemed to only slow cell growth and suppress PSA expression, but paradoxically resulted in more metastases [76] from xenografts in immunocompromised mice.

Given the difficulties of working with an industry standard cell line, which carries a mutated AR target gene, a number of investigators transfected new copies of wild-type AR into LNCaP [77]. These exogenous AR copies are not under normal cellular expression control, and expression is frequently driven by strong heterologous promoters, which do not display the self-regulatory nature of the human AR transcriptional control sequences. However, the synthetic cells (termed generically LNCaP/AR) were ultra-responsive to the addition of DHT, as judged by PSA production, and have been used in the biological testing of the next-generation drugs such as enzalutamide [78,79]. More recently, the LNCaP/AR cells were used to elucidate the mechanism by which SOX2 overexpression influences resistance to enzalutamide [80], which was developed after analyses of human tissues and in a mouse prostate knockout model [81]. An observed lineage plasticity, and requirement to lose or inactivate tp53 and pRb1, was common between the models, resulting in a basal/NE-like resistant cell type in which inhibition of the master transcriptional controller EZH2 restored sensitivity to ADT. However, derivation of enzalutamide-resistant cells from four different prostate cancer cell lines (including LNCaP but not LNCaP/AR) was unable to replicate the SOX2-based mechanism [82]. These ENZA^r^ cells also failed to show an increased growth dependence on glucocorticoids [82], previously reported to be another primary resistance mechanism [83]. There are further multiple reports of NE-like cells after serum starvation [84] or epigenetic manipulation of LNCaP [85] —in the absence of any ADT/enzalutamide treatments.

We and others have proposed the existence of low/no PSA expressing cancer cells with an aberrant basal phenotype [86,87] both in vitro and, perhaps more importantly, in cells freshly extracted from human prostate cancers [88]. Their cancer origins are defined by the presence of changes to PTEN status [89], overexpression of c-myc, the presence and expression of TMPRSS2-ERG fusion genes [90] and a cancer-like CpG methylation profile [91]. These cells are naturally unresponsive to the blockade of androgen signaling and would provide a reservoir of resistant cells which produce tumor-regenerating cells either by the acquisition of inactivating mutations or at the cessation of the AR inhibitory treatment.

Does this range of responses reflect the sustained ability of the LNCaP model to (i) establish in culture from an original xenograft, (ii) an ability to survive changes in laboratory culture conditions or (iii) the development of a “convenient” fast-growing laboratory clone? When one now considers the history of LNCaP—40 years of culture (since 1978) initially in growth medium containing 15% FCS, selection for a fast growing clone, changes in karyotype (4 X chromosomes in 1979; now, only one AR copy in the FGC clone) a median chromosomal number of 85-89 and an extreme phenotypic plasticity based on culture conditions [70,85,92]—LNCaP does allow the study of AR gene regulation, but is it still the ideal model (in 2020) on which to base studies of the precise molecular mechanisms of prostate cancer AR response and treatment resistance?

### 3.3. Cell Death in Animal Models of PCa after Androgen Signaling Blockade

In animal models, the first biological evidence for prostate cell death after castration came from the response of normal tissue to surgical castration [93], where the rat prostate rapidly involutes, and both morphological and molecular evidence of apoptosis was evident. In a follow-on from this work, the same techniques plus a determination of specific gene expression changes (to TGF beta, TRPM2 and calcium signaling) were shown in xenografts of the androgen-sensitive human PC82 cancer xenograft. In this model, the peak of apoptotic signaling was achieved after 72 hours, accompanied by tumor cell death, and a 70-fold decrease in Ki67 staining, indicative of a complete blockade of cell replication, was noted [94]. 

In direct contrast, a similar study in rats, using the highly differentiated Dunning R3327PAP tumor, detected no rapid reduction in tumor cell number at 3, 7 and 14 days post-surgical castration [95]. The Dunning system of rat tumors has been exploited for many years since its development [96]. Whilst it offers an in vitro/in vivo shuttling experimental model with a wide range of responses to androgens and castration [97], its relevance to human disease is probably limited by the low homology between rodent and human prostates, both morphologically and in the effective concentration of androgens circulating in the experimental animals—all of which contribute to the inability of the Dunning tumors to secrete recognized human prostate cancer markers [98]. 

In the Dunning R3327PAP cancer cells, whilst the mitotic index was reduced after castration, no morphological or molecular evidence of apoptosis was seen. However, similar apoptotic cell death to that seen in normal rat prostate was seen in the adjacent normal prostate [95].

### 3.4. Cell Death in Human Tissues after Androgen Signaling Blockade

In human tissue, there seem to be major differences in castration responses compared to the experimental animal and cell line studies. Murphy et al. [99] examined the morphological consequences of ADT (leuprolide +/– flutamide) in 12 sequentially sampled patients. After 2–14 months (median 3–4 months) of treatment, neither degeneration nor necrosis in the neoplastic prostate glands but an increase in the inter-glandular stromal cells was seen, and the authors hypothesized that the effect of ADT was to arrest tumor growth, rather than induction of apoptotic or any other mechanism of cell death. Interestingly, they noted that ADT affected only certain cell populations within the cancer mass, unlike the response of the more homogeneous PC82 human PCa xenograft, where widespread cell death was observed [94]. Whilst serum PSA was essentially zero in these ADT-treated patients, a persistent PSA protein signal in the prostate tissues was observed.

A larger study using 18 patients 3 months after castration [100] observed some apoptotic cell death, mostly in the lowest grade tumors (probably now reclassified as abnormal rather than cancerous under the amended Gleason grading system [101]), but reported that the majority of patients showed (as in Murphy et al. [99]) both tumor cell atrophy and a decreased proliferation rate. It should be recalled that at 3 months post-castration, PSA levels in patients have probably only decreased by around 30%, at a time point long after that used in the animal xenograft studies (Section 3.3).

In conclusion, the many cell line studies (including those in the industry standard LNCaP cells and variants thereof) promote the case for an active cell death based on apoptosis, either in vitro or in xenografts. This certainly looks to be the case in normal human tissues, but no such effect is seen in human cancers. The expansion of human stroma is intriguing and demonstrates the importance of considering the entire prostate response to castration, rather than the convenience of fast-growing cell lines.

## 4. Gene Expression Changes in Prostate Epithelial Cells during the Application of an Androgen Signaling Blockade in Patients

**Figure 5:**
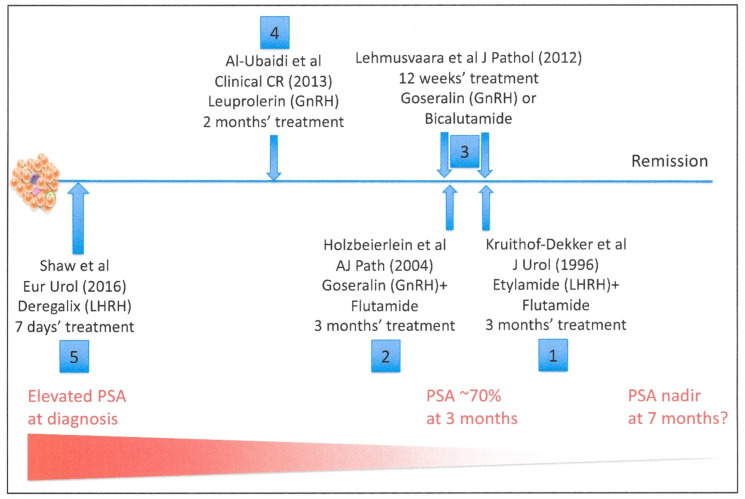
Gene expression studies after androgen blockade in prostate tissues. The total numbers of up and downregulated genes after ADT are shown in green and red, respectively, in Table 2.

The human tissue data for cell death are therefore both incomplete and inconsistent with the simplified view provided by the cell line and animal model systems. Since it is the human disease which should drive treatment strategies, a number of studies have approached mechanisms of tumor death by studying gene expression in directly biopsied human tissues. The timing of the various studies with respect to treatment initiation are extremely variable and are summarized in Figure 5. Androgen-based therapies (ADT) do have remarkable effects on prostate cancer growth and progression. However, it has also been shown that the AR-stimulated transcriptome in cancer cells is quite distinct from that in non-malignant cells [102], which complicates any study of unfractionated human tissue where normal cells frequently coexist.

To monitor the effects of ADT in patients, prostate-specific antigen (PSA) remains an excellent biomarker of AR activity in both normal and malignant cells [103]. As a biomarker of cancer treatment in man, a 30% decrease in PSA expression is seen after 3 months androgen blockade and an absolute PSA nadir at 7–8 months [104]. This is not comparable to the effects of a strong cytotoxic drug in rapidly growing cells, where evidence of immediate cell death and tumor shrinkage (within 72 hours) can normally be seen—the time point used in many of the cell line model studies. However, remarkably little is known about the biology and genetics of the treatment in man, beyond its effects on PSA.

### 4.1. The Dynamic Changes in Gene Expression after ADT in Human Tissues

In early gene-specific studies (Figure 5 and Table 2), designed to test a specific hypothesis, Kruithof-Dekker et al. [105] showed by immunohistochemistry that there was a surge in the expression of estrogen receptor (ER alpha) after 3 months of treatment with etylamide + flutamide (the equivalent of total androgen blockade in 1996). More recently, Al-Ubaid et al. [106] detected an upregulation of the DNA repair protein Ku70 after 32 months ADT using leuprolerin, perhaps implicating DNA damage responses as part of the ADT effect on target cancer cells [107]. The two processes are increasingly associated, given the successful clinical use of poly ADP-ribose polymerase-1 (PARP) inhibitors as prostate cancer therapies [108], but in the subset of patients with DNA repair deficiencies.

A better examination of the genetic consequences of ADT comes from studies of whole genome transcriptomes, initially by Holzbeierlein et al. [109] (goserelin + flutamide) and updated by Lehmusvaara et al. [110] (goserelin + bicalutamide). Both studies used patient tissues biopsied after 3 months of treatment. The expression changes gave no indication of the mechanisms of cell death, which surely should have been apparent at a time point when PSA levels were falling in the patients.

A common restriction in such gene expression studies (of mRNA) is the timing of the studies. Biological effects are frequently preceded by changes in mRNA expression over hours or even days. For this reason, a more recent study by Shaw et al. [111], which was carried out on patient biopsies after 7 days of deregalix treatment, could have provided more informative data about the primary tissue responses to ADT. Whilst no evidence of a cell death mechanism was again evident, a strong upregulation of estrogen receptor was seen, but localized to the stromal cells, rather than epithelial cells. This was in broad agreement with the (3 month) ADT immunohistochemical studies of Kruithof-Dekker et al. [105]. However, the two studies differed in the cellular location of any epithelial ESR expression; whereas the earlier IHC showed expression in sporadic basal cells, Shaw et al. [111] found expression in luminal (AR+) cancer cells.

Despite remarkably similar working protocols, the total gene expression studies revealed only a few genes whose expression was commonly altered in all of the studies. There were irregular increases in the expression of a number of genes implicated in alternative AR stimulation pathways, such as IL6 [112], but also other genes which mark stem-like and amplifying normal cells in the prostate, such as SMARCD [113], ALDH [114] and ETS2 [115]. However, the only gene whose expression was increased in all three transcriptional studies was retinoic acid responder 1 gene (RARRES1)—a known tumor suppressor gene in prostate [116], whose expression is positively regulated by retinoic acid in differentiating normal skin epithelium [117]. My own laboratory has identified RARRES1 as a transcript repressed in the tumor-initiating population of prostate epithelial cells but upregulated in the tissue, restoring the population of (normal) basal transit amplifying cells [88,118]. Thus, the expression signature for ADT-treated prostate tissues (from all three studies) at 3 months after treatment initiation probably reveals not a cell death profile but rather a very rapid increase in tissue repair, initiated by a normal basal cell population and probably regulated by retinoids. In both the Prostate Cancer Prevention Trial (PCPT) [119] and in a meta-analysis of 15 other studies [120], there was a positive correlation between serum retinoid levels and poor survival in prostate cancers. An orphan retinoic acid receptor (RORgamma) can also drive the AR response program in the absence of AR or its ligand in CRPC [121]. Whilst a regenerative RA response is marked strongly by RARRES1 upregulation, regrowth of the tumour component is less likely to be marked by increased RARRES1 expression, due to the uniformly low levels of RARRES1 detectable in most cancer cell types, including prostate [116,118].

A more recent update of the responses to androgen blockade, left as an imprint in the circulating tumor cells (CTCs) from patients, was revealed by a single cell RNA sequencing study of CTCs from 13 patients [122], although the intact cell numbers studied were low (77) since most such CTCs are probably inviable or undergoing apoptosis. Such a focused study of persistent changes could also alter our thinking about the nature of CRPC in man. The data emphasized the importance of alternative splicing of the AR to yield a ligand-independent activation [123] but also revealed a lower than expected frequency of changes in expression and mutation of the glucocorticoid receptor gene, previously reported to be a primary mechanism of enzalutamide resistance [83]. Therefore, either the CRPC CTCs are a different tumor subclone to that seen in biopsy material and in mouse xenografts [124], or we are dealing with an evolving cell type in man (as shown in below).

### 4.2. The Dynamic Changes in Gene Expression after Castration in Mouse Tissues

To better model the effects of castration, animal models allow the sequential sampling which is not possible in human patients. Accordingly, the indeterminate cell types, which expressed the genes showing expression changes in the human experiments, can be resolved by unbiased single-cell RNA sequencing and cluster analysis to identify specific cell types. Recently, Karthaus et al. [125] reported such a single-cell RNA sequencing analysis to identify cellular subpopulations in mouse prostate. They were able to identify three different luminal populations, as well as a basal population. When the mice were castrated, a proportion of the luminal cells survived and were responsible for prostate regeneration (a rapid event in mouse prostate). RNA signature comparisons with human prostate samples identified a number of similarities, but not identity between the murine luminal cells and human luminal epithelium. All of the biology was carried out on mouse systems, and functional associations to man were drawn by comparisons of similarity. Conclusions about prostate regeneration after ADT were made on the same basis. However, this study provides a real indication that we now have the means to answer the key questions about the mechanism of ADT (and resistance development).

The currently published evidence does not yet give a clear picture, even when studying gene expression changes in human tissues. Perhaps significantly, no evidence of cell death gene expression was seen at a time when serum PSA levels are falling in patients, but rather the activated genes indicate either a persistence of unaffected tissue or even tissue regeneration.

## 5. Modeling Resistance to Androgen Blockade: The Quest for a Defining Mechanism

The main issues with the study of tumor resistance (in man) will probably only be resolved by analysis of multiple biopsies taken before, during and after ADT relapse. Many patients are unwilling to be subjected to biopsy while in remission, so most studies are again carried out in cell lines and animal models—or as end point data in relapse/autopsy (after perhaps several months or years of treatment). Should such biopsies be obtained, the choice of biopsy site in a late-stage, highly heterogeneous population of tumors could be compromised by the genetic heterogeneity and lack of a known tumor development lineage [126]. Thus, unbiased single-cell sequencing [125] should be the method of choice, were the clinical material made available. It is also important at this stage to recall the caveats previously discussed concerning mechanisms of responses in the various model systems when examining the nature of relapse from ADT.

### 5.1. Cell Line Studies of the ADT Resistant State

Most established cell lines can be manipulated to ADT resistance—for example, by culture of LNCaP for up to 12 months in increasing concentrations of enzalutamide to generate EnzaR cells for further study, which mimics the extended exposure times of ADT patients in vivo, and the selective pressure to develop such resistant cells. The molecular basis of resistance is nevertheless complex, involving both changes to the AR gene (which are extensively discussed elsewhere [28,29,126,127,128,129] and were summarized in Table 1).

Some of these molecular mechanisms are perhaps not unexpected, if the nature of the androgen signaling pathway is taken into account. There are three possible outcomes of such pathway manipulation. Firstly, the removal of exogenous androgens by LHRH prevents the flow of substrate (hormone) into the pathway. The system can react by activating new and endogenous means of synthesizing substrate (androgens) to compensate [130]. When the response pathway is inhibited (as desired), it also reacts by providing a greater pool of the receptor to react with as much of the ligand which is remaining. This can occur at several levels: (i) simple overexpression of the AR gene [31,131]—a more reversible response to the changing microenvironment, (ii) alternative splicing of the AR mRNA to generate a truncated protein which can translocate to the cancer cell nucleus and activate gene expression in the absence of DHT ligand [33,127,128] or (iii) activation of a non-androgen-driven salvage pathway, as illustrated in Figure 2, but also by utilization of the glucocorticoid receptor (GR) protein [124]. In multiple AR-expressing cell line models, blockade of retinoic acid receptor-related orphan receptor γ (RORgamma) reduced cell viability, by suppressing its ability to initiate AR stimulated gene expression programs in the absence of the ligand [121,129]. The duality of control for many genes with AR and other retinoic acid receptors [132] in prostate epithelial cells provides further evidence of a stem-like nature of CRPC, revealed not only in gene expression and micro-RNA expression patterns, but also provides a direct link to the single cell CRPC CTC sequencing studies discussed earlier where genes such as WNT5A [88] and ALDH7A1, CD44 and KLF4 were overexpressed in 60% of the heterogeneous CRPC CTC populations [65]. Alternatively, mutagenic changes occur in the cancer cell genome, which are irreversible and leave the cells susceptible to further treatment strategies (see below) such as (i) specific mutations within the AR gene to broaden the substrate choice for the receptor, (ii) amplification of the AR gene, as seen in up to 50% of prostate cancers, as either X chromosome aneuploidy or more commonly a tandem amplification of the AR locus (see Table 1).

### 5.2. Complexity and Heterogeneity: Modeling ADT in Three Dimensions

A major issue with cell line models is their inability to recapitulate the exquisite interplay seen between the epithelial and stromal components of the human prostate. A key role for “tumor stroma” has been proposed for many years [133,134,135] to affect the invasive capacity of the epithelial component of the cancer. This is carried out by the AR-expressing component of stroma and is therefore likely at least in non-reactive (non-tumor) stroma to be susceptible to the inhibitory effects of anti-androgens [136]. However, the AR-induced transcriptomes of epithelial and stromal cells are also quite distinctive [137]. Perhaps paradoxically, recent data have implied that the effects of testosterone are repressive in stroma (unlike epithelial cells) [138], a biological result identified by our own studies on prostate cell recombinations in synthetic matrix [139]. There is also no doubt that the transcriptomes seen in vitro have a number of distinct differences from those seen in 3D tissues, mainly concerned with cell cycle (cultured cells favor growth rather than homeostasis in tissues) and the maintenance of telomeres [140].

To model this, tissue reconstructions have been employed [141]. These do more closely mimic responses in vivo but do require the correct constituent of matrix and stromal cells (of an early passage in culture after surgical biopsy). More recently, this technique has been augmented by the emergence of tissue organoids (reviewed by Wang, Gao and Chen [142]). Single-cell expression profiling [125] identified a population of androgen-sensitive mesenchymal/stromal cells to which was tentatively assigned a regenerative, post-castration function to supply essential growth factors to the regenerating epithelial cells. This inductive function is reminiscent of embryonic prostatic organogenesis [143], where the stromal cell origin determined the ultimate differentiation of epithelial precursors into vestigial prostate glands in my own laboratory, generation of polarized and functional (PSA secreting) human adult glandular prostatic epithelium (from an initially AR-basal cell population of primary human cells) which required the presence of both androgens and androgen-sensitive stromal cells [141] to complete the process.

Organoid tools were originally designed to study self-renewal in murine and human tissue stem cells [144] and have been reported in prostate, particularly of murine origins [145,146]. However, the efficiency of organoid generation from human prostates is much more efficient from normal prostate and is highly selective from human prostate cancers [145]. The underlying danger with all of these techniques is their very selectivity. Clonality of cells in a “model system” results in more consistent data for pathway inhibition, for example, but tumors (particularly prostate) are heterogeneous. Investigators assume that the cells which grow in culture are those which drive oncogenic growth in vivo and often erroneously assume that the majority of the cells in an organoid are those which present the major therapeutic target. Most organoid systems also lack the in vivo input from the carcinoma-associated fibroblasts, as described earlier. In the single-cell sequencing experiments [125], the murine organoids generated did not include the AR-responsive mesenchymal cells that they described. Future studies employing controlled multicellular human organoids and single-cell phenotyping after ADT in three dimensions in vitro could resolve some of these issues, including those of mouse:human relevance and the mechanisms of cell death and regeneration. Most importantly, even these experiments should only provide hypotheses for subsequent testing in human tissues.

### 5.3. ADT in Genetically Engineered Mice

In a review of mouse models [147], the importance of selecting the most appropriate model to answer a specific biological question was stressed. The authors concluded (like many before) that no model was perfect, but a recent publication which describes mice with a multiple gene deletion core has achieved a closer concordance with human disease and its response to androgens [148]. When these mice were castrated at 13–15 weeks and dosed with enzalutamide for 3 weeks, the tumors did not progress over a 3–5-week period, after which a tumor which mimics CRPC emerged and all animals died by 35 weeks (20 weeks after treatment initiation), compared to untreated mice which died within 25 weeks. Only limited evidence was presented on mechanisms, however.

Castration has also formed the basis for the discovery of putative luminal precursor cells in a PTEN/KRas model of mouse prostate cancer—the (castration-resistant Nkx3.1 expressing) CARN cell [149]. The AR gene has subsequently been experimentally deleted in this cell population [150], after which the ability of the mice to form tumors was only somewhat compromised, and small populations of cancer cells with NE differentiation developed, similar to those seen in a proportion of patients.

### 5.4. Human Xenografted Cell Models

Unlike the GEM models, which retain an intact mouse immune system, xenografted human prostate cancer cell lines usually produce relative structureless tumors, some of which retain expression of human AR protein [151]. A good example of an exploitable PDX model is the PC346 androgen-responsive xenograft [152]. More commonly, cell lines are treated in vitro with escalating doses of ADT drugs, followed by regrafting into the murine host. The rapid growth kinetics of the cell lines in mice makes long-term castration experiments difficult to carry out with these established tumors, which also lack the input from human tumor stroma in most models—where the stromal component is normally from the mouse host.

Better models which are closer to the original patients (patient-derived xenografts or PDXs) are frequently, in their initial stages, slow growing whilst also retaining expression of active AR [153], especially after chemical boosting of the (low) levels of circulating androgens in the mouse host. However, it remains a challenge to establish such PDX from hormone-naïve patients.

## 6. Modeling Pathway Responses to Androgen Signaling Blockade

**Figure 6:**
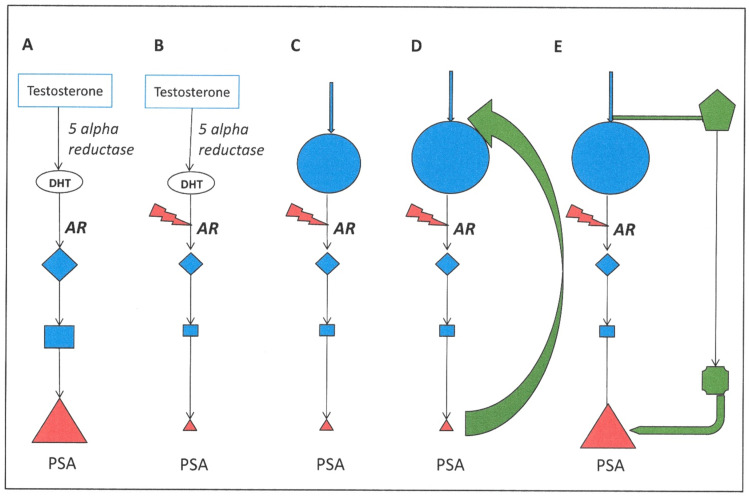
Theoretical pathway inhibition after direct blockage of AR activity. Whilst most clinical emphasis is on the downstream effects (biomarkers such as PSA) of AR inhibition (**B**), the accumulation of signaling molecules before the blockade can have profound metabolic consequences (**C**). This is achieved not only by accumulation before the drug blockade, but also by feedback stimulation of flow into the pathway (**D**). Ultimately, the excess of substrate can be relieved in the cell by activation of a salvage pathway (**E**), which results in a restoration of output (PSA) by other means.

The very nature of interlinked signaling pathways in cells, and an empirical focus on the “expected outcome” of enzyme inhibition, can result in speculation about both unexplained and poorly understood consequences. Figure 6 illustrates the theoretical consequences of a blockage in the AR signaling pathway—for example, by a small molecule (Column 1). The inhibition would result in the expected suppression of PSA expression (for example), but also results in an accumulation of substrates before the blockade (Column 2). The natural consequence of loss of the end product (such as PSA) from a pathway is feedback to generate an even greater accumulation of substrates (Column 3). At this point, most pathways activate a salvage or relief pathway to remove the unwanted excess of signaling intermediate molecules (Column 4).

This results in the accumulation of progesterone and selection of cells containing adaptations of the AR by mutation [154,155].

**Figure 7:**
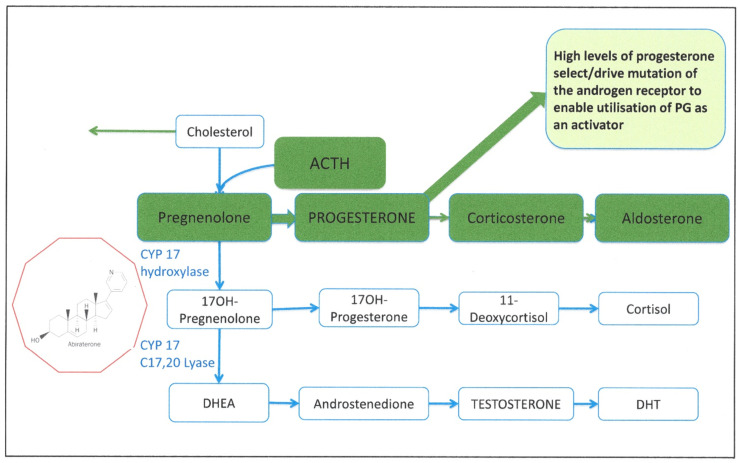
Accumulation of steroid precursors after inhibition of CYP17 by abiraterone. DHT: Dihydroteststerone, DHEA: dehydroepiandrosterone, ACTH: Adrenocorticotropic hormone.

A clinical example of this is shown in Figure 7, where the testosterone biosynthetic pathway has been effectively inhibited by abiraterone action to block CYP17 hydroxylase and CYP17 C17, 20 lyase enzymes. Under these conditions, there is an accumulation of pregnenolone and the pathway to aldosterone production is favored. This is in perfect agreement with an observed escape mechanism—frequent mutation of the AR gene (T878A) to utilize progesterone as an activator molecule [154,155]. The same mechanism can be inferred for resistance to estramustine (in LNCaP cells, for example), where estrogen became an activator of the mutated receptor (T877A) [68]. Lastly, even enzalutamide, highly engineered to strongly bind to and inhibit AR protein [129], exhibits this metabolically-based resistance, displaying a relaxation of AR substrate specificity to include glucocorticoids via a F876L mutation [124,156]. It is not known whether subclones with such AR mutations pre-exist in a heterogeneous tumor mass (intrinsic resistance) or are indeed induced by the drug treatment.

However, this is a simplified scheme, as there are probably multiple and simultaneous activations of such salvage pathways in real tumors. Artificial intelligence approaches should permit anticipatory modeling of these alternatives, perhaps even to allow secondary treatments.

## 7. Combination Drug Treatments: Is More Better?

In the ideal therapeutic strategy, the clinician would be able to anticipate the resistance and block it by targeted combinations as and when it arises—a more dynamic form of ADT. This probably requires a superior biomarker to PSA. However, in the case of ADT, there are so many alternatives that, unless the resistance is corralled into a single pathway, even combination therapies with attendant risks of off-target effects are unlikely to succeed in the longer term. Current trials of dual AR inhibition (e.g., abiraterone plus enzalutamide), whilst effective in the short term in the PLATO trial, showed little or no benefit for the combined treatment of men with chemotherapy-naïve metastatic CRPC [157] and may simply be a better way to achieve total androgen blockade, which, despite its early promise, resulted in inevitable relapse [158,159].

Of course, a key limitation would be if different clones within the same tumor mass activated different AR salvage pathways simultaneously upon application of ADT (induced resistance) in genetically unstable prostate cancer cells. This would be the oncological equivalent of fighting a wildfire, where new secondary fires are constantly being established. Rationally, a novel approach could be to direct the resistance towards a particular pathway and include a secondary treatment for the favored escape mechanism simultaneously with the initial ADT.

## 8. Cellular Heterogeneity and Resistance to Androgen Blockade: Is Resistance Intrinsic or Induced?

### 8.1. Do Prostate Cancer Stem Cells Provide a Treatment-Resistant Reservoir?

What can we ultimately learn from detailed genomic and transcriptomic studies of CRPC in human tissues when compared to the hormone-naïve cancers from which they are presumably derived? Unfortunately, only rarely are tumors from the same patient subjected to such analysis, and there remains considerable doubt about just which cells serve as the origin for CRPC [149,159,160,161]. Depending on the experimental system, many explanations have been proposed, such as the following:

- trans-differentiation from an AR +/sensitive tumor cell to AR+ CRPC [161,162];
- expansion of an AR ± but androgen insensitive population of CAR cells [149]; or
- the presence of a no/low AR-expressing stem-like cell population in the primary tumors [87,88].

The existence of this minor population of stem-like cells remains controversial. There are many instances of identification in established cell lines, which has implied that these rare stem-like cells are an artefact of cell culture. However, recent studies using cells extracted directly from tissues and analyzed immediately without culture have indicated the presence of progenitor-like cells with a unique cancer phenotype as predicted from colony forming ability in cells from cancer tissue extracts [163]. In mice there is, as previously discussed, evidence for more luminal cells (Section 5.1, Section 5.2 and Section 5.3) in prostate tissue regeneration [125,149].

The emergence of stem-like cells, with increased expression of developmentally associated regulatory pathways, has also been noted in cells resistant to enzalutamide. For example, as discussed earlier, WNT signaling [65] is detected after ENZA treatment, and elevated NOTCH signaling is also seen within 4 hours of the start of enzalutamide treatment in vitro [64]. Rather than indicating increased cell replication, malignancy or tumor progression, NOTCH expression may act to preserve a population of stem-like cells, as seen, for example, after radiotherapy treatments in glioblastoma [164] and human prostate [165].

As we learn more about genomic sequences in the cancer types, and the heterogeneous nature of even primary prostate cancers, we are less certain to conclude that a patient’s CRPC is the product of adaptive evolution from the originally diagnosed (and treated) primary tumor. For example, a proportion of hormone-naive prostate cancers include mutations in the isocitrate dehydrogenase gene (IDH1). Such mutations are, however, absent in almost all of the CRPC tumors sequenced to date [166]. Similarly, the frequency of the diagnostic TMPRSS2-ERG fusion in hormone-naive prostate cancers is greater than that found in poorer prognosis and ultimately CRPC, perhaps by as much as twofold [167]. In contrast, other mutations such as PTEN deletion on chromosome 10q 21 are common across the disease spectrum, in fact increasing in frequency in CRPC [166].

### 8.2. Evidence for Pre-Existing Resistant Cells in Human Clinical Trials

**Figure 8:**
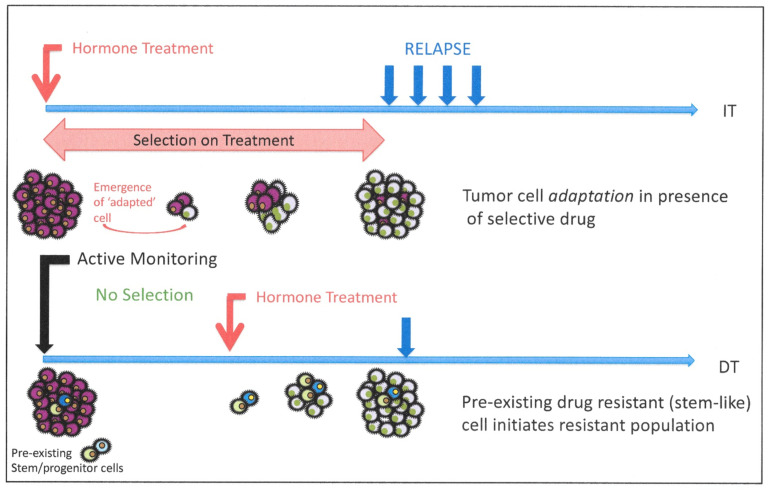
Prior treatment with ADT in a human clinical trial does not induce resistance. In a human clinical trial [168], the results were best explained by a selection for pre-existing resistant cells, rather than induction by the presence of the AR inhibitor (IT = Immediate Therapy; DT = Delayed Therapy).

There has been at least one clinical trial [168] which approached the topic of pre-existing or induced resistance in patients. In 2006, an EORTC trial (30891) reported on outcomes of immediate or deferred hormone therapy in patients with prostate cancer unsuitable for local treatment with curative intent. The trial outcomes are illustrated in Figure 8.

Significantly, in contrast to most other studies at that time, their endpoint was not an increase in serum PSA, but rather the onset of “clinical symptoms and progression of hormone refractory disease”. This would be less acceptable in contemporary studies, but the use of image-driven relapse detection (in the presence/absence of increased PSA) could now offer similar outcome data to update this study. Even with a relatively imprecise means of determining clinical relapse, remarkable overlap in the relapse curves was observed in a trial of 985 randomized patients comparing immediate vs. deferred ADT. The data did reveal a modest (10%) survival advantage for immediate hormone treatment (compared to delayed) over the 11-year follow-up (225/492 vs. 209/493). Were the immediate application of AR therapies inducing a new cell population, one would expect a more rapid relapse rate upon treatment failure, due to an increased frequency over the time of treatment, of resistant cell clones in the cancer. The close coincidence of the relapse curves does imply the presence of a pre-existing resistant population, acting as the “seed” for relapse. Similarly, it has been possible to detect small “nests” of cells with AR copy number increases in hormone-naïve prostate cancers in the absence of any ADT induction [30].

Although beyond the scope of this review, the origins of CRPC relapse towards, for example, a neuroendocrine phenotype, which is now more common as a result of secondary ADT, are also open to speculation.

**Figure 9:**
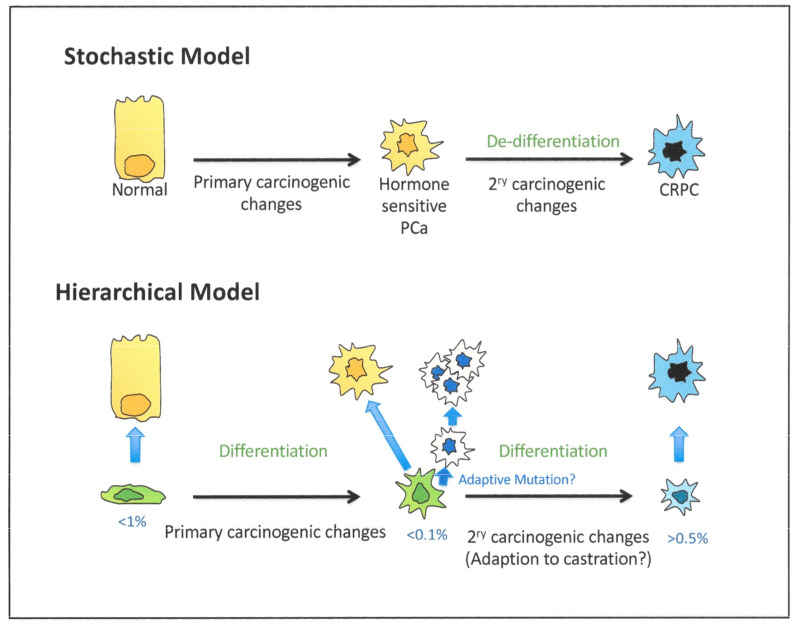
Models for development of castration-resistant prostate cancers. Upper Panel: In a trans- or dedifferentiation model of resistance, the tumor cells are growth arrested by the presence of the AR inhibition. During growth arrest, the tumor cells have a genetic plasticity which pushes tumor cells towards a drug-resistant phenotype by the presence of the drug. Most tumor cells can therefore be the progenitors for the resistant (CRPC) tumor, and the CRPC cells will share most if not all of the mutations in the original bulk tumor cells. Lower Panel: In a hierarchical or stem cell model of resistance, there is a small population of relatively undifferentiated (or stem-like) cells present in every tumor, which contain cancer driver mutations. Under selective pressure from an anti-AR drug, which arrests the growth of the bulk cancer cells, variants can emerge from the common pre-existing precursor which develop new adaptive mutations. Assuming that the original bulk cancers had developed adaptive mutations from their underlying progenitors, the resultant CRPC cells should share only the driver mutations with the original cancers and have a new set of changes for growth under ADT conditions.

As illustrated in Figure 9, the hypothesis of an adaptation (or dedifferentiation) of a CRPC phenotype to neuroendocrine-like tumors has been proposed in both patients and in animal models [160,169]. Rare NE-like cells exist in normal prostate [170], although recently, their origin in normal mouse prostate gland development has been suggested to be the neural crest, instead of a common prostate precursor cell [171]. Minor populations of rare NE tumor cells are also seen in HDT-naïve cancer tissue sections [170] amidst the hormone-responsive tumor mass, increasing in number upon development of CRPC. However, the mutational profiles do not exclude the presence of rare, treatment-resistant, pre-existing, less differentiated cells, which can amplify and aberrantly differentiate to generate both the neuroendocrine-like cancers and more common glandular CRPC [126]. However, NE-like cells can be generated by epigenetic manipulation of normal and malignant epithelial cells, whose markers of origin they retain, so there is a developmental precedent as well as an invocation of a novel trans-differentiation process. A resolution of this would pave the way for a better treatment of the currently treatment refractory NE tumor types.

## 9. Modeling Pathways to CRPC—Predictions from Mechanism Testing

Without the ability to study human tumor development in vivo (in real time), it will remain almost impossible to distinguish between the two most credible alternative mechanisms which result in castration-resistant disease (Figure 8), i.e., stochastic or hierarchical changes. Perhaps the best resolution of the alternatives will lie in the use of selective inhibitors. The process of trans-differentiation from a luminal-like cell to CRPC will almost certainly require activation of a different geneset from a more conventional or stalled differentiation of a stem-like androgen-insensitive (basal-like) precursor to the stem-like phenotype of CRPC. However, we should remember that the starting cell type from which CRPC derives is a tumor cell, with known phenotypic plasticity and an underlying number of patient-specific variable driver gene changes [172] required to achieve a tumor phenotype from a normal/premalignant precursor. It has proved difficult to extract a “standard” tumor phenotype from current prostate cancer gene expression databases [166].

## 10. Does Better Androgen Blockade Change the Natural History of Prostate Cancer?

### 10.1. Long-Term Effects of Low Androgen Levels in Men with Benign and Malignant Prostate Disease

Hundreds of thousands of men are treated every year with increasingly potent ADT drugs, all designed to block the androgen signaling axis in prostate cancer cells. The clinical effects are immediate, the biomarkers (PSA, for example) are easily monitored and tumor masses are reduced—albeit by an undetermined mechanism. ADT is a major healthcare expenditure, but we still do not know at a molecular level the short term, or even more significantly, the longer-term effects of persistently low levels of AR signaling in men. As stronger ADT is applied more generally and perhaps even at an earlier disease stage in more men, the adverse effects may become more apparent—but are these valid in the face of evidence of increased short-term efficacies? For example, does long-term application of ADT result in a change in the natural history of prostate cancer—for example, more small-cell basal-like and neuroendocrine tumors [173], which are notoriously difficult to treat? Changes in the locations and types of ADT-resistant metastases are also emerging, where “soft tissue” lesions are increasing in prevalence [174].

As discussed above, the low levels of cell death after ADT in patients, and the persistence of viable ADT-resistant cells rather than their elimination, provides the ideal breeding ground/selection system for variant development. We know that this happens, since CRPC will develop in the majority of patients—we just do not know how it happens [126,160,161,162].

Therefore, does the strategy of generating new ADT agents and a focus on the sequencing of the various blockades of the AR signaling pathway offer patients the best chance of long-term treatment? The cycling of ADT [15,175], and/or the perhaps anti-intuitive approach of overstimulation of the AR pathway [176,177], offer a remission but, nevertheless, temporary survival advantages. Application of ADT does offer an opportunity for more curative combinations of treatment, however. The achievement of growth arrest and regrowth in a cyclic application would seem to offer the best opportunity to eliminate minor resistant populations.

### 10.2. Androgens Are Not Just Active in Male Reproductive Organs: Extra-Prostatic Effects of Long-Term ADT

As a male reproductive organ, the high level of expression of the AR in cells from the prostate and its disorders, such as benign prostatic hyperplasia (BPH), prostatic intraepithelial neoplasia (PIN) and prostate cancer, is not a surprising experimental result. However, AR expression is not restricted to the secretory, epithelial component of prostate tissues [178]. Conversely, there are epithelial cells in prostate which do not express detectable AR protein [179]. In mouse prostate development, it is the androgen response not of the epithelial component but of the stromal cells which promotes initial prostate epithelial differentiation and organogenesis [180], as shown in the embryonic mesenchymal+epithelial cell mixture experiments of Cunha et al. [143], which clearly showed that the embryonic fate of the epithelial cells was largely determined by AR-driven stromal signals.

Therefore, androgen response inhibition in man should effectively limit the growth and development of the prostate and testes as a minimum, but is this a truly tissue-targeted therapy? The 5-alpha reductase inhibitors such as dutasteride or finasteride showed variable efficacy as either chemopreventive or treatment agents for prostate cancer—revealed in a much-debated enhanced detection of high Gleason score cancers in the PCPT trial [26], whilst apparently suppressing low-grade cancers [181]. More recent studies [182] have reinforced the positive aspects of the PCPT trial output, although we remain naïve about the best time and age to apply such therapies in men. The perceived adverse effects resulted in the rejection of 5-alpha reductase inhibitors by the FDA in a chemoprevention role for prostate cancer, and similar studies with the dual-action 5-alpha reductase inhibitor Dutasteride in the subsequent REDUCE trial failed to show any effect on more aggressive tumors whilst shrinking lower grade or faster growing cancers (summarized by Schroder and Roobol [183]). Hormonal interventions in most Gleason score 6 and lower cancers, when organ confined, may even be unnecessary, given the data from recent active surveillance trials such as PROTECT [184].

However, one interesting “off-target effect” of 5-alpha reductase inhibition (e.g., with Propecia) has been hair regrowth in cases of male pattern baldness, resulting in a repurposing of the latter drugs by the manufacturers. However, such long-term use of hormone activity manipulators seems to be having undesirable effects in a number of health areas, including but not limited to libido [185]: around 15,000 post-finasteride adverse effects at a clinically relevant level have been reported to an online forum (pfsfoundation.org), perhaps implying a more widespread relevance of androgen signaling in man than just in the prostate, although the recent re-evaluation of the PCPT trial largely refuted a finasteride-based mechanism for these effects [182]. As the number of patients on long-term maintenance of prostate cancer remission with ADT increases, more evidence accumulates about potential physical effects of androgen suppression, including decreases in bone mineral density, metabolic changes such as weight gain, decreased muscle mass, and increased insulin resistance, decreased libido and sexual dysfunction, hot flashes, gynecomastia, reduced testicle size, anemia, and fatigue [186].

An increase in cardiovascular events has been frequently noted, but statistically, the evidence for this remains marginal [187], although increased death due to cardiovascular events is regularly observed.

**Figure 10:**
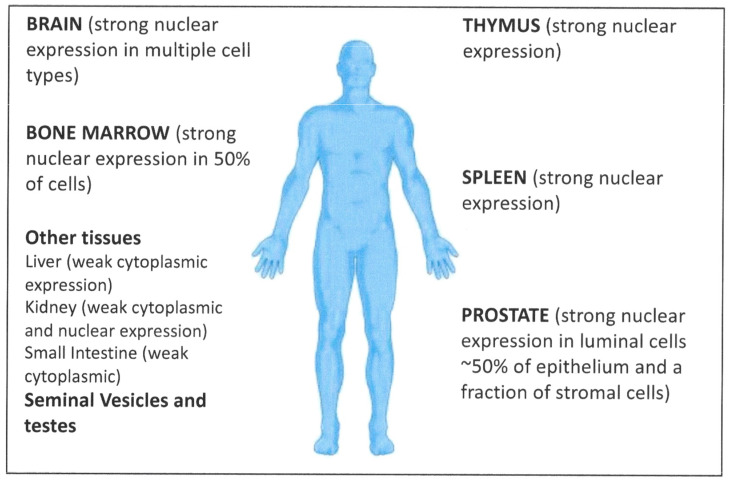
Expression patterns of human androgen receptor in multiple and non-prostatic tissue data from Dart et al. [178] and other sources in text.

Adverse effects on animal brain development with finasteride treatment have recently been reported in rat brains [188] and, as summarized in Figure 10, active AR signaling (indicated by high levels of the protein in the cell nuclei) has been observed, for example, in the brain [189]. A more widespread distribution of AR mRNA and protein is recorded in the online gene expression databases. Whilst strong expression is indeed seen in the male reproductive tract, similarly strong expression of AR protein in the cell nucleus was more markedly detected in bone marrow, spleen and thymus [178,190]. There are currently a number of (tissue) selective androgen receptor modifiers (SARMs) available [191]. Is it too great a leap of faith or chemistry to redesign these tissue-specific reagents as inhibitors? Exploitation of tissue-targeting SARMs could also provide the advantages of AR supplementation to muscle-wasting diseases and immune depletion in patients undergoing treatment with the current AR inhibitors.

Most current and next-generation AR inhibitors can cross the blood–brain barrier (with the reported exception of Darolutamide (Bayer) which is currently in clinical trials (ARAMIS [192]), but whether brain expression of AR is directly linked to observed seizures, as an adverse effect of therapy, remains unproven [193].

In the immune regulatory tissues, it is credible to expect that the cytoproliferative and protective effects of AR expression would be immune-stimulatory and that the observed depletion of such responses could provide an alternative explanation for the relatively poor responses of patients to prostate immunotherapy, since most such treatments are being employed after failure of ADT [194]. Paradoxically, however, in castrated (or AR knockout) rodents, the size of the thymus actually increases and is reduced by the inoculation of testosterone [190]. Indeed, given such widespread expression of the target (AR) molecule, it might be questioned whether androgen receptor inhibition would today be licensed de novo for use in any but the most terminally ill cancer patients!

## 11. Future Perspectives: Exploiting Mechanistic Biology for Patient Advantage in An-drogen-Based Therapies

If we accept that the effects of ADT in man do differ from those in animal models and in vitro cultures, longer-term and precise monitoring studies during clinical trials will be required to unlock the resistance mechanisms. The advent of single-cell gene expression analysis is likely to identify better secondary targets than more detailed genomic sequencing, as there is increasing evidence that the first responses of human cancer cells to therapy are at an epigenetic level, in, for example, pancreatic cancers [195]—a lower mutation cancer type, similar to pre-ADT prostate cancers. The presence of higher numbers of mutations in CRPC tissues at autopsy is probably indicative of developmental changes as the tumor grows to a final fatal form. As discussed above, “prostate cancer” and “CRPC” are both generic terms describing a tumor state, which can be achieved by many pathways. Single-cell sequencing, just like the multiple mechanisms of resistance to ADT, provides the best case for stratified or even patient-specific treatments based on individual tumor genome/epigenomes. However, which cells should be sequenced? I would promote analysis of ALL viable cells ahead of recognizable “prostate” or “epithelial cells”, all too frequently based on AR expression. To reject a cell phenotype which does not confirm to an expected type might result in the exclusion of a rare precursor of treatment failure—a lesson to be learned from studies of fetal brains and brain tumors, where “new” cell subtypes can be recognized after single-cell expression profiling [196].

## 12. Conclusions

Identification of the prostate tissue cells in which ADT stimulates regenerative gene expression, whether normal or malignant, should be a priority in order to make a very good time-limited palliative treatment into an excellent curative one. The keys to achieving lasting benefits from ADT lie in the prompt identification and blockade of the secondary pathways by a directed combination therapy, before a resistant clone of new tumor cells emerges either by a trans-differentiation /selection or from a pre-existing pool of resistant stem-like cells, for example. With the current evidence, I am unable to answer the question posed in my title—there is evidence for both pre-existing and induced resistance, although the results are critically dependent on the model used for study. However, even the state of the art in hormonal therapy is time-limited and resistance does indeed seem inevitable.

Androgen-based therapies are not inevitably doomed to failure: they are highly effective in the short term. Perhaps exploitation of the range of agents now available in a more biologically determined fashion would offer better long-term outcomes for more prostate cancer patients.

## Figures and Tables

**Table 1:** Androgen receptor signaling by conventional and alternate mechanisms can be inhibited by specific small-molecule inhibition.

Drug Class	Molecular Target	Drug Example	Escape Mechanism	Ref.
**Androgen synthesis: serum testosterone levels**	Pituitary gonadotrophin secretion. Luteinizing hormone releasing hormone agonist (LHRH)	Zoladex	Intratumoral androgen synthesis AR gene amplification to utilize low intratumoral androgens	[19,20,21]
**Androgen synthesis**	Intratumoral CYP17	Abiraterone	Use of glucocorticoid receptor and glucocorticoids	[22,23,24]
**Testosterone activation to dihydrotestosterone (DHT)**	Steroid 5-alpha reductase	Dutasteride/Finasteride	Switch in 5aR isotype or use of testosterone/adrenal androgens	[25,26]
**Androgen Receptor (AR) inhibitor**	DHT binding to monomeric androgen receptor	Steroidal: Estrogen, Cyproterone acetate Nonsteroidal: Casodex, Enzalutamide	Gain of function mutations in the androgen receptor gene to enable it to use other steroid hormones. Bypassing of the AR by ligand independent activation of androgen responsive genes	[27,28,29,30,31,32]
**Dimerization of AR**	Androgen receptor	Stilbene, Resveratrol	Mutation of the AR gene and expression of ligand-independent splice variants	[33,34,35]
**Phosphorylation of androgen receptor**	AR S81: CDK1, CDK5 and CDK9 AR S213, S791 and T850: PIM1 and Akt AR S213: CXCR4	Roscovitine, 5,6-dichloro-1-b-D-ribo-furanosylbenzimidazole (DRB) and flavopiridol SGI-1776, TP-3654 (PIM1) Miltefosine, Perifosine, MK-2206 and GSK-2141795 (Akt)	Redundancy in kinase usage	[36,37]
**Nuclear translocation of androgen receptor**	Androgen receptor	CH5137291, Diallyl Trisulfide Enzalutamide	Expression of AR splice variants which translocate to the nucleus in the absence of androgen.	[38,39]
**Binding of nuclear androgen receptor to: (1) Recognition sites on DNA;** **(2) Co-activator molecules**	Androgen receptor and co-activators	AR DNA Binding Domain: 4- (4-phenylthiazol-2-yl) morpholine AR interaction interface: LXXLL/FXXLF motifs	Changes in balance between AR co-activator and co-repressor molecules and relative affinity of the receptor by mutation of AR.	[40,41,42]
**Downstream effects of proteins stimulated by AR activity**	Androgen response molecules	Multiple	Activation of alternative salvage pathways which stimulate the signaling molecules downstream from AR activation.	[43,44,45,46]
**AR stability and degradation**	Heat shock proteins (HSP90) Serine proteases, caspases and calpain	LAQ824, a cinnamyl hydroxamatic acid histone deacetylase inhibitor 17-allyamino-17-demethoxygeldanamycin (17-AAG) Multiple	Redundancy in the heat-shock chaperone system Inhibition of AR proteolysis	[47,48,49,50,51]

The data in the table are not intended as an exhaustive list but are an illustration of the many escape mechanisms available to a prostate cancer cell from ADT.

**Table 2:** Gene expression studies in human tissues after ADT.

Study	Treatment	Molecular Outcomes	Year
1	3 months Etylamide (LHRH)+Flutamide	21 patients by immunohistochemistry–only studied (estrogen receptor alpha) ESR1: Intense Stromal ESR+ and normal sporadic basal cells, NOT in CaP cells. No Ku70 expression	1996 [105]
2	3 months Goseralin (GnRH)+Flutamide	Transcriptional study: (290/364 *) AR-regulated genes repressed. No ESR changes (one gene) but SMARCD, ETS2, IL6, ALDH and **RARRES1** stimulated. AR expression increased in CRPC only. No Ku70 expression.	2004 [109]
3	12 weeks Goseralin (GnRH) or Bicalutamide	Transcriptional study: (B 97 and G 62) and (B+G 89) changed by >2 fold. 24/128 genes directly AR regulated. Some overlap (16%) within study but little with others, no ESR1 changes, but **RARRES1** upregulated. No KU70.	2012 [110]
4	2 months Leuprolerin (GnRH)	Studied DNA damage repair after treatment specific for Ku70 and gamma H2AX. Ku70 decreases with castration mirroring PSA but no grade-specific changes.	2013 [106]
5	7 days Deregalix (LHRH)	Initial transcriptional analysis (749/908). Estrogen receptor upregulation (E+S) in cancers + normal basal cell gene expression (**RARRES1**). No KU70.	2016 [111]

* Upregulated genes shown in green, downregulated in red. The only gene significantly upregulated in all 3 ‘total genome’ transcription studies was the retinoic acid responder 1 gene (RARRES1).
